# The Presence or Absence of Intestinal Microbiota Affects Lipid Deposition and Related Genes Expression in Zebrafish (*Danio rerio*)

**DOI:** 10.3389/fmicb.2018.01124

**Published:** 2018-05-29

**Authors:** Yi Sheng, Hui Ren, Samwel M. Limbu, Yuhong Sun, Fang Qiao, Wanying Zhai, Zhen-Yu Du, Meiling Zhang

**Affiliations:** ^1^Laboratory of Aquaculture Nutrition and Environmental Health, School of Life Sciences, East China Normal University, Shanghai, China; ^2^Department of Aquatic Sciences and Fisheries Technology, University of Dar es Salaam, Dar es Salaam, Tanzania; ^3^Key Laboratory of Exploration and Utilization of Aquatic Genetic Resources, Shanghai Ocean University, Ministry of Education, Shanghai, China

**Keywords:** germ-free, lipid metabolism, zebrafish, gut microbiota, antibiotic

## Abstract

Understanding how intestinal microbiota alters energy homeostasis and lipid metabolism is a critical process in energy balance and health. However, the exact role of intestinal microbiota in the regulation of lipid metabolism in fish remains unclear. Here, we used two zebrafish models (germ-free and antibiotics-treated zebrafish) to identify the role of intestinal microbiota in lipid metabolism. Conventional and germ-free zebrafish larvae were fed with egg yolk. Transmission electron microscopy was used to detect the presence of lipid droplets in the intestinal epithelium. The results showed that, microbiota increased lipid accumulation in the intestinal epithelium. The mRNA sequencing technology was used to assess genes expression level. We found majority of the differentially expressed genes were related to lipid metabolism. Due to the limitation of germ-free zebrafish larvae, antibiotics-treated zebrafish were also used to identify the relationship between the gut microbiota and the host lipid metabolism. Oil-red staining showed antibiotics-treated zebrafish had less intestinal lipid accumulation than control group. The mRNA expression of genes related to lipid metabolism in liver and intestine was also quantified by using real-time PCR. The results indicated that *apoa4*, *hsl*, *cox15*, *slc2a1a*, and *lss* were more related to intestinal bacteria in fish, while the influence of intestinal microbiota on the activity of *fabp6*, *acsl5*, *cd36*, and *gpat2* was different between the liver and intestine. This study identified several genes regulated by intestinal microbiota. Furthermore, the advantages and disadvantages of each model have been discussed. This study provides valuable information for exploring host-microbiota interactions in zebrafish in future.

## Introduction

The intestine as the most important absorptive organ is densely colonized with microbial cells referred to as microbial community (Costello et al., 2009). The microbial community is considered to be a metabolic “organ" in the host (Backhed et al., 2004) that ferments indigestible dietary polysaccharides (Zhang et al., 2014), increases lipid accumulation in adipocytes (Turnbaugh et al., 2006), and influences nutrient absorption and energy balance (Semova et al., 2012).

Triacylglycerides (TAGs) are the main energy sources in animals, but the potential effect of the intestinal microbiota on host lipid metabolism has not been thoroughly studied (Semova et al., 2012). Increasing evidences suggest the close relationship between the gut microbiota and the lipid metabolism. For example, a bacteria isolated from an obese human gut combined with high-fat diet (HFD) induced germ-free mice obesity and insulin resistance which may also be caused by lipopolysaccharide (LPS) (Fei and Zhao, 2013; Yan et al., 2016). Compared to conventional mice, germ-free C57BL/6J mice gained less weight and accumulated less fat pad when fed a chow or HFD (Backhed et al., 2004; Rabot et al., 2010). In contrast, the colonization of adult germ-free mice with bacteria harvested from the cecum of conventional animals resulted in a significant increase in body fat content (Backhed et al., 2004). All these findings indicated that intestinal microbiota participated in the host lipid metabolism.

To date, identifying the role of intestinal microbiota has been mainly limited to mammals, which represent less than 10% of total vertebrate diversity (Sullam et al., 2012). To further unveil the functions of intestinal microbiota, it is critical to expand our understanding on the role of intestinal microbiota on aquatic animals (Zhang et al., 2016). Zebrafish (*Danio rerio*) has been used to explore the role of microbial community on immune system regulation, development, and nutrient acquisition (Semova et al., 2012; Rolig et al., 2015; Hill et al., 2016). Except for the genetic and genomic resources, zebrafish has several advantages over mammals, including high fecundity rate, rapid external development, and optical transparency. Zebrafish larvae initiate exogenous feeding at approximately 5 days post-fertilization (dpf) and complete resorption of their endogenous yolk supply by 6 dpf (Semova et al., 2012). Hundreds of zebrafish can be easily derived and maintained in a germ-free state (Pham et al., 2008); therefore, zebrafish is a suitable model animal for the analysis of intestinal microbiota and host physiology (Leulier et al., 2017).

Germ-free animal model has been successfully used to identify the role of intestinal microbiota in mammals (Backhed et al., 2004; Fei and Zhao, 2013; Ruiz et al., 2017). However, studying nutritional mechanisms by using germ-free zebrafish still has many defects. For example, the life span of germ-free zebrafish is too short to be suitable for nutritional analysis, and accordingly it is not easy to identify the phenotypic and metabolic characteristics of different organs (Pham et al., 2008). Antibiotics treatment commonly depletes intestinal microbiota. Therefore, antibiotics-treated animals are another popular model for exploring the function of commensal microbiota (Brandl et al., 2008; Ruiz et al., 2017). It is convenient to detect the influence of commensal bacteria in liver or intestine and identify the underlying molecular mechanisms by using antibiotics-treated animals (Morgun et al., 2015; Ruiz et al., 2017). However, antibiotic-induced effects can be explained by depletion of the microbiota, direct effects of antibiotics on the host or the effect of remaining antibiotic-resistant bacteria (Morgun et al., 2015). Therefore, care must be taken when antibiotics-treated animals are used to identify the functions of commensal microbiota.

In this study, two models, including germ-free and antibiotics-treated zebrafish, were used to assess the influence of intestinal microbiota on lipid accumulation and related genes expression in zebrafish. Furthermore, advantages and disadvantages of each model in nutritional and metabolic analyses in zebrafish were also compared.

## Materials and Methods

### Ethics Statement

All experiments were performed under the Guidance of the Care and Use of Laboratory Animals in China. This research was approved by the Committee on the Ethics of Animal Experiments of East China Normal University (ECNU) (No. F20140101).

### Production of Germ-Free Zebrafish

Adult male and female conventionally raised zebrafish were cultured in a recirculating zebrafish aquaculture facility at ECNU. The production of germ-free zebrafish was performed as previously reported (Pham et al., 2008) with some minor modifications. Briefly, fertilized eggs were washed three times by using sterile water (3 min per cycle at room temperature) and incubated for 4 h at room temperature in 50 mL of gnotobiotic zebrafish medium (GZM) which contained 0.06 mg/mL marine salt (Hai Ye, Shanghai, China), 100 μg/mL ampicillin, 5 μg/mL kanamycin, and 250 ng/mL amphotericin B. Zebrafish embryos were subsequently rinsed three times using GZM (3.5 min per cycle at room temperature), immersed in 0.04% polyvinylpyrrolidone for 40 s, rinsed three times by using GZM at room temperature, and immersed in 0.002% sodium hypochlorite (Sangon Biotech, Shanghai, China) for 15 min at room temperature. They were further rinsed three times using GZM and reared in a sterile 25-cm^2^ flask with sterile (autoclaved) GZM at a density of approximately 0.4 individual per mL water. A total of 50% by volume of GZM in each dish was replaced with fresh medium on daily basis during the experiment. Three replicates for each well were used to test the sterility of the culture water on a daily basis using a tryptic soy agar (TSA) plate (Pham et al., 2008). Briefly, 100 μL of media from each well was spotted onto a TSA plate. The plates were cultured aerobically at 28°C for 24 h. The sterility of the culture water was also tested by using Luria-Bertani (LB) plate at 28°C for 24 h. Water temperature was maintained at 28°C with an external PRX-80 Intelligent Incubator System (Sai Fu, Ning Bo, China). Three flasks of conventional and germ-free zebrafish were fed sterile egg yolk for 3 h at the 7 dpf resulting into the fed conventional zebrafish (CF) and the fed germ-free zebrafish (GF) groups, respectively. Six individual whole zebrafish from each treatment were weighed and homogenized in 1 mL phosphate-buffered saline (PBS). A total of 100 microliter of the homogenized mixture was plated in LB plates for bacterial count.

### Antibiotics-Treated Zebrafish Model

A popular cocktail of antibiotics was used to deplete the intestinal bacteria according to a previous study (Brandl et al., 2008). Vancomycin (CAS:1404-93-9), neomycin sulfate (CAS: 1405-10-3), and metronidazole (CAS:443-48-1) were purchased from Yuanmu Biotechnology Co., Ltd., Shanghai, China. Unfortunately, there was no study during the experimental period that has established the antibiotics concentrations for depletion of commensals in fish intestine. Therefore, three different diets were formulated that contained three concentrations of antibiotics as follows: MNV1 (metronidazole 2 g/kg diet, neomycin sulfate 2 g/kg diet, vancomycin 1 g/kg diet); MNV2 (metronidazole 4 g/kg diet, neomycin sulfate 4 g/kg diet, vancomycin 2 g/kg diet); and MNV3 (metronidazole 8 g/kg diet, neomycin sulfate 8 g/kg diet, vancomycin 4 g/kg diet). The formulation and composition of the diet is shown in Supplementary Table S1. During the experiment, fish were hand-fed a diet with or without antibiotics at a ratio of 4% of their average body weight twice daily for 3 days. At the end of the rearing period, the intestinal content from six individual zebrafish in each treatment were homogenized in 1 mL PBS. A total of 100 microliter of the homogenized mixture was plated in LB plates for bacterial count. A control group (CN) containing zebrafish reared without antibiotics treatment and antibiotics-treated group (AN) containing zebrafish treated with antibiotics were involved in this experiment.

### Transmission Electron Microscopy (TEM)

Transmission electron microscopy (TEM) was used to monitor the lipid droplets (LDs) in the intestinal epithelial. Five zebrafish larvae were fixed with 2.5% glutaraldehyde and 1% osmium tetroxide for 2 h at 4°C after 7 dpf. Samples were washed six times by using PBS for 2 h at room temperature, dehydrated three times with graded acetone (from 30% to 100%), and embedded in pure Epon812 epon-based resin. Ultrathin sections (60 nm) were obtained using an ultramicrotome equipped with a diamond knife and collected on copper grids. The ultrathin sections were stained by using 1% uranyl acetate for 20 min and with lead citrate for 8 min. The stained sections were observed under a transmission electron microscope (HT7700, Hitachi, Japan). LDs accumulation was assessed in a 5-μm-wide area in epithelial cells close to the lamina propria. All micrographs were analyzed using Software ImageJ (1.4.3.67).

### Oil-Red Staining

Oil-red staining was performed to identify the lipid accumulation in the intestine. Three fish from control and antibiotics-treated groups were collected. Approximately 0.5-cm-length segments of the foregut were gently flushed with PBS and incubated at 4°C twice overnight, first in 4% paraformaldehyde and then in 30% sucrose. A 10 μm of intestinal sections were obtained and stained by using oil-red O and hematoxylin. Stained slides were observed under microscopy (Nikon, Eclipse, TS100).

### RNA Extraction and Illumina RNA-Seq

In order to detect the genes expression, total RNA was extracted from 20 conventional or germ-free zebrafish larvae using Trizol (Invitrogen, Carlsbad, CA, United States). The RNA purity was determined by electrophoresis in 0.8% agarose gels and its concentration was determined by using a Nanodrop 2000 (Thermo Fisher Scientific, Waltham, MA, United States). The RNA was purified by using poly-T oligo-attached magnetic beads and fragmented for the synthesis of complementary DNA (cDNA) libraries. For each sample, 2 μg cDNA was used for libraries’ construction by using TruSeq RNA sample preparation kit (Illumina, San Diego, CA, United States) and sequenced in Hiseq Illumina platform (Illumina, San Diego, CA, United States) using the following primers: F: 5′-ACACTCTTTCCCTACACGACGCTCTTCCGATCT-3′ and R: 5′-CGGTCTCGGCATTCCTGCTGAACCGCTCTTCCGATCT-3′. The conditions for sequencing consisted of one cycle at 98°C for 2 min 30 s followed by 10 cycles at 98°C for 15 s, 65°C for 30 s and 72°C for 30 s and lastly 72°C for 5 min. Paired-end reads with the length of 150 bp were obtained.

### Data Filtering and *de Novo* Assembly

The raw data collected were inspected to remove unsuitable reads for performing *de novo* assembly. The adapter sequences and reads with low quality scores (< 20) or a final length < 50 bp were discarded. FastQC^1^ was used to assess the quality of the raw data. TopHat^2^ was used to map the RNA-Seq reads compared to zebrafish GRCz10. Transcriptome raw data from this manuscript can be obtained from the National Center for Biotechnology Information (NCBI) Sequence Read Archive under accession No. SRP106934.

### Gene Annotation

To extend the evolutionary genealogy of genes, non-supervised orthologous groups – eggNOG^3^ – were used to identify and annotate gene distribution (Powell et al., 2012). Expression level of each gene was calculated by reads per kilobase per million (RPKM) in Perl scripts by normalizing the sequence coverage over the gene length and total unique mapped reads in the library (Mortazavi et al., 2008). Differentially expressed genes (DEGs) were identified by using HTSeq^4^ and DESeq^5^. The DEGs were defined as genes showing at least a 2.0-fold change in transcription expression level based on Bayesian statistical analysis. Kyoto Encyclopedia of Genes and Genomes (KEGG) is a database that is used to determine the high-level functions of biological systems, such as metabolism, cellular process, and organismal systems (Ogata et al., 1999). We used KEGG Mapper to identify statistically significant enrichments of DEGs in KEGG pathways (Kanehisa et al., 2004). DEGs associated with nutrient metabolism and RPKM extreme deviation > 4 were selected for further heatmap analysis. The relative proportion (RPKM/RPKM median) of DEGs in each group was calculated. The |Log_10_ (relative proportions)| was used to distinguish upregulated (|Log_10_ (relative proportions)| > 0) while the (|Log_10_ (relative proportions)| < 0) depicted the downregulated DEGs. The cluster analysis was performed using MATLAB R2016a.

### Quantitative Reverse Transcription-PCR

To quantify the genes expression levels or validate the RNA-seq data, total RNA from six zebrafish samples for each treatment was extracted using Tri Pure Reagent (Aidlab Biotechnologies Co., Ltd., China). Only total RNA having an A260/A280 absorbance ratio of 1.8–2.0 and an A260/A230 ratio > 2.0 was used for subsequent analysis. Electrophoresis was also used to assess the RNA quality by visualizing the 28S/18S ribosomal RNA ratio on a 1% agarose gel. The RNA quantity was measured by using Nanodrop 2000 Spectrophotometer (Thermo Scientific, MA, United States). The cDNA was synthesized by using 1 μg total RNA as the template by a PrimeScript^TM^ RT Reagent Kit (Takara, Dalian, China) according to the manufacturer’s instructions. The primers used for qRT-PCR in this study are presented in Supplementary Table S2. The qRT-PCR was performed in a CFX96^TM^ Real-Time PCR Detection System (Bio-Rad, Hercules, CA, United States) with SYBR Green qRT-PCR Mix (Bio-Rad, Bio-Rad, Hercules, CA, United States). The qPCR mixture contained 10 μL of 2 × SYBR qPCR Mixture (Takara, Dalian, China), 100 ng cDNA, 300 nM of qPCR primers, and 6.4 μL nuclease-free water. The qPCR conditions consisted of one cycle at 95°C for 30 s followed by 40 cycles at 95°C for 5 s and an annealing step at 60°C for 20 s. The melting curves of the amplified products were analyzed at the end of each qPCR. Each qRT-PCR run was performed in triplicate and negative controls (no cDNA) were conducted. The expression level of each gene was normalized against the expression level of the eukaryotic translation elongation factor 1 alpha 1 (Ef1α). The relative expression levels were analyzed by 2^-ΔΔ^*^C^*^t^ and fold changes were calculated between CF versus GF and CN versus AN.

### Statistical Analyses

Statistical analyses were performed by using Prism 5 (GraphPad Software Inc., San Diego, CA, United States). Differences in LDs number, bacterial number, or gene expression level between the conventional and germ-free zebrafish and control and antibiotics-treated zebrafish were detected by using Student’s *t*-test with subsequent Bonferroni’s correction. Differences with *p*-values < 0.05 or 0.01 were considered statistically significant or highly significant, respectively.

## Results

### Effects of the Intestinal Microbiota on Lipid Absorption in Germ-Free Zebrafish

**FIGURE 1:**
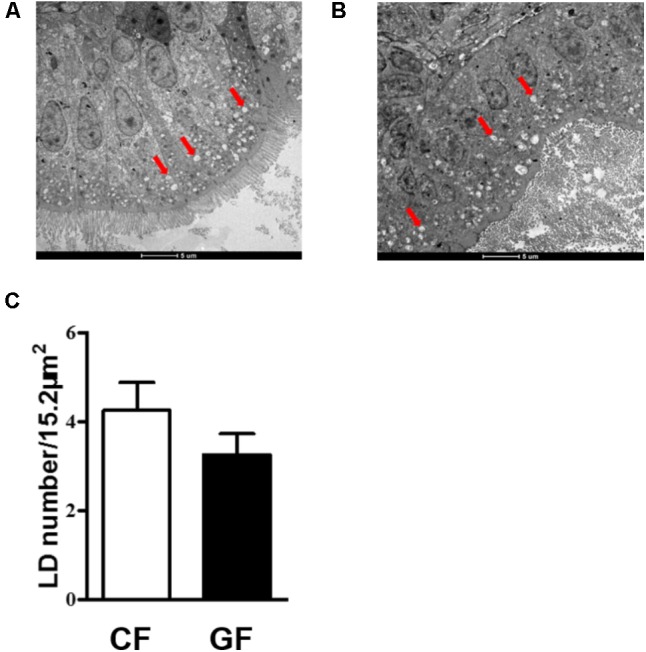
TEM images of lipid droplets (LDs) in the intestinal epithelium of conventional zebrafish fed egg yolk **(A)**, germ-free zebrafish fed egg yolk **(B)**, the number of LDs in 15.2 μm^2^ intestinal epithelium was calculated **(C)**. Red arrows indicate the LDs. Data are presented as mean ± SEM. Significant difference was determined by Student’s *t*-test, *p* < 0.05. The magnification of the TEM is 1200×.

Before the treatment, LB plate was used to detect the bacterial quantity of germ-free and conventional zebrafish. The results indicated that no any bacteria were detected in the germ-free zebrafish and the bacterial quantity was about 10^6^ CFU/g for conventional zebrafish. To assess the effects of the intestinal microbiota on lipid absorption, we counted LDs number in the intestinal epithelial of conventional or germ-free zebrafish on the 7th days (**Figures 1A,B**). Considering epithelial LDs were detected after 3 h incubation with egg yolk, the number of LDs of about 5-μm-wide epithelial section was counted in each group based on at least three TEM images. The results indicated that LDs number was comparatively higher in conventional fish than in germ-free fish. However, no significant difference was detected (**Figure 1C**).

### DEGs in Conventional and Germ-Free Zebrafish

Illumina sequencing of CF and GF cDNA libraries generated approximately 35.82 and 32.88 million paired-end reads, respectively, with an average length of 150 bp. The quality value ≥ 20 with more than 94.85% bases for each sample and the quality value ≥ 30 with more than 90.82% bases for each sample suggested that the sequencing results were reliable. After removing low-quality sequences (quality value < 20) and adaptor sequences, a total of 35.29 and 32.42 million clean reads were generated for CF and GF groups, respectively (Supplementary Table S3). Guanine and cytosine bases (GC) content for CF and GF were 54.85 and 53.23%, respectively. Additionally, 80.96 and 96.26% of the reads were mapped as unique sequences in the CF and GF, respectively (Supplementary Table S3).

**FIGURE 2:**
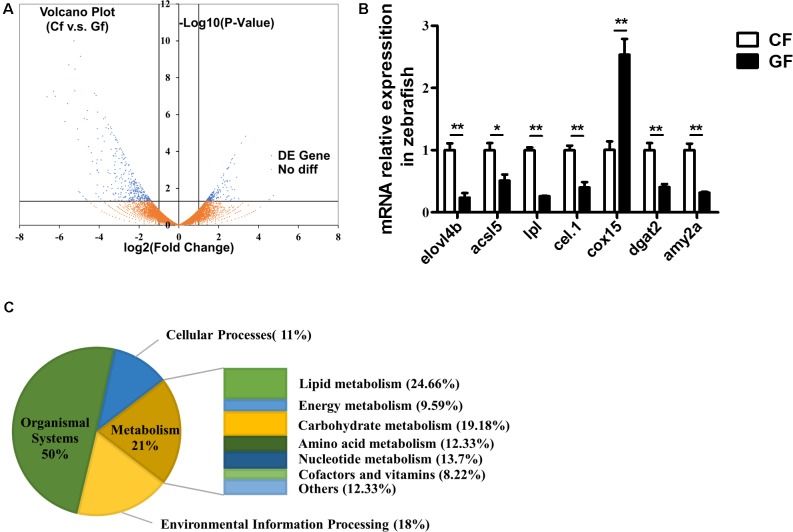
RNA-Seq data generated from zebrafish including conventional or germ-free zebrafish. **(A)** Volcano plot of differentially expressed genes (DEGs) from the transcriptomes of CF and GF. For each unigene, the ratio of expression levels (CF versus GF) was plotted against the -log error rate. The horizontal line indicates the significance threshold (FDR, 0.05), and the vertical lines indicate the twofold change threshold. Non-DEGs are shown with orange dots and DEGs are shown with blue dots. **(B)** The distribution of differently expressed genes according to KEGG enrichment analysis related to nutrient metabolism, including lipid metabolism, carbohydrate metabolism, nucleotide metabolism, amino acid metabolism, energy metabolism, cofactors and vitamins, and other amino acid metabolism. **(C)** Real-time PCR validation. Data are presented as mean ± SEM. The expression levels normalized to CF. Significant difference was determined by Student’s *t*-test, ^∗^*P* < 0.05; ^∗∗^*P* < 0.01.

A Volcano plot indicating DEGs between the conventional and germ-free zebrafish is shown in **Figure 2A**. The dots located in the positive and negative areas represent 164 and 366 genes expressed higher in GF and CF, respectively. DEGs between CF and GF were attributed into four groups according to KEGG enrichment analysis. The groups included organismal system (50%), cellular processes (11%), environmental information processing (18%), and metabolism (21%) (**Figure 2B**). In the present study, we focused on the genes involved in metabolism which contained lipid metabolism (24.66%), carbohydrate metabolism (19.18%), nucleotide metabolism (13.7%), energy metabolism (9.59%), amino acid metabolism (12.33%), cofactors and vitamins (8.22%), and others (12.33%) (**Figure 2B**).

To validate the RNA-Seq results, 27 genes related to lipid metabolism or energy harvest were randomly selected for real-time PCR validation (**Figure 2C** and Supplementary Figure S1A). The results confirmed the validity of RNA-Seq and indicated that expression of *elovl4b*, *acsl5*, *lpl*, *cel.1*, *dgat2*, *amy2a*, *gpat2*, *cd36*, and *apoa4* decreased significantly, while *cox15* and *lss* increased significantly in GF compared with the CF ones.

### Effects of the Antibiotics Treatment on Lipid Accumulation and Metabolism

**FIGURE 3:**
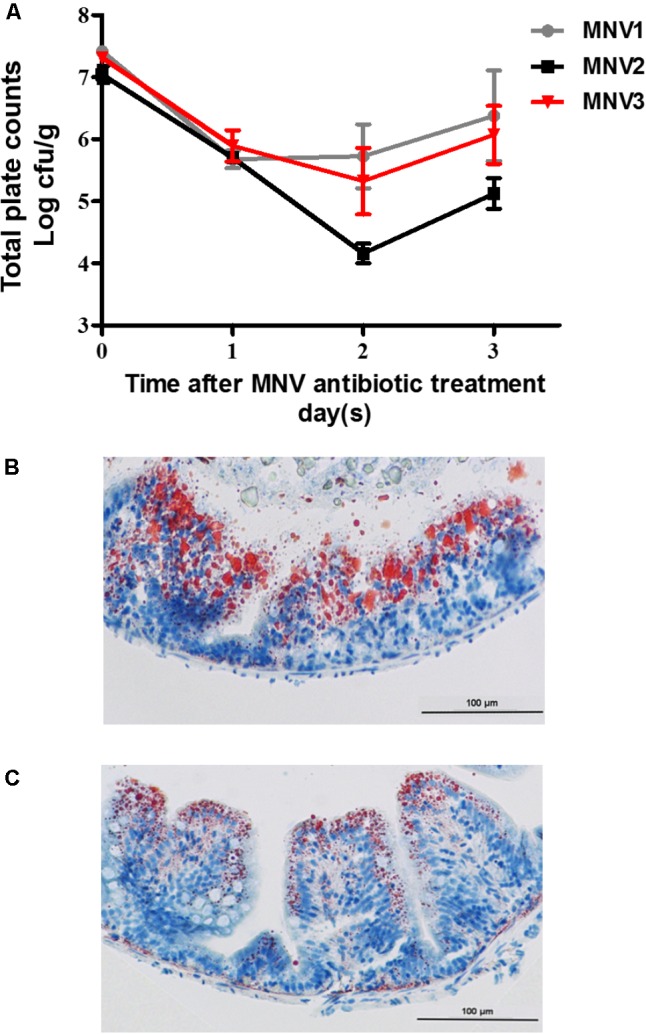
Influence of antibiotic treatment on bacterial load and lipid accumulation in fish gut. **(A)** Change of bacterial quantity with different concentration of antibiotic treatment for 3 days. Oil-staining of lipid accumulation in control **(B)** and antibiotic-treated group **(C)**. The magnification is 400×.

To construct the antibiotics-treated zebrafish model, the effect of antibiotics on the intestinal microbiota was evaluated. Different dietary concentrations of antibiotics mixtures were fed to zebrafish for 3 days and the bacterial quantity was detected on daily basis. The results indicated that a marked decrease in total bacterial quantity per gram of intestinal content was obtained after the 2nd day on zebrafish fed on MNV2 treatment (**Figure 3A**). The intestinal tissues from the CN and AN fed on MNV2 treatments on the 2nd day were collected for further oil-red staining to show the accumulation of lipid. The results indicated that compared to CN, AN group had less LDs (**Figures 3B,C**). Genes which have been previously detected in GF in this study were detected in the liver or gut of CN and AN groups. The genes expression for *cyp51* and *fabp6* increased significantly while *cd36* and *apoa4* decreased significantly in the liver of AN group compared with the CN group (Supplementary Figure S1B). In the intestine, *cox15*, *pparγ*, and *slc2a1a* increased significantly while *atgl*, *fabp6*, *acsl5*, and *apoa4* decreased significantly in AN group compared with the CN group (Supplementary Figure S1C).

## Discussion

### Genes That Responded to the Presence of Bacteria in Both Models

The detected genes can be divided into three clusters (**Table 1** and Supplementary Table S4). Five genes, including *apoa4*, *hsl*, *cox15*, *slc2a1a*, and *lss* showed the same trend to the presence of intestinal microbiota in both models. It has been found that *apoa4* is involved in integrating feeding behavior, intestinal lipid absorption, and energy storage (Wang et al., 2012). The expression of *apoa4* was regulated by LPS and therefore promoted the production of high-density lipoprotein (Dandekar et al., 2016). The expression level of *apoa4* was higher in conventional zebrafish than germ-free or antibiotics-treated fish, verifying the close relationship between *apoa4* and the intestinal bacteria. We found the expression level of hormone-sensitive lipase *(hsl)* was higher in conventional zebrafish than germ-free or antibiotics-treated ones (Supplementary Figure S1). Hormone-sensitive lipase activities of the epididymal adipose tissue were higher in conventional than in germ-free rats (Kawai et al., 1981), consistent with our present study. Although we focused on lipid metabolism, we also selected some genes related to other nutrients metabolism. For example, *slc2a1a*, a member of solute carrier family which is responsible for glucose transporter, was upregulated in germ-free or antibiotics-treated zebrafish in the present study, suggesting that without the intestinal microbiota, animals need to increase energy intake (Swartz et al., 2012). Another gene, *Lss* encodes lanosterol synthase, which increased in germ-free or antibiotics-treated zebrafish in the present study, suggesting that the absence of microbiota promotes cholesterol loss and a compensatory upregulation of mechanisms controlling cholesterol synthesis in germ-free animals (Zhong et al., 2015).

**Table 1:** Comparison of expression patterns observed with two models.

	CF/GF^a^		CN/AN^a^			
			Liver		Intestine	
Gene^b^	Fold change^c^	*p*-value	Fold change^c^	*p*-value	Fold change^c^	*p*-value
*apoa4*	4.80	0.055	3.91	0.037*	10.72	0.003**
*hsl*	1.95	0.207	1.56	0.218	2.64	0.067
*dgat2*	2.46	0.009**	1.32	0.327	1.54	0.352
*slc2a1a*	0.57	0.176	0.54	0.207	0.40	0.009**
*lss*	0.59	0.04*	0.72	0.635	0.46	0.233
*cox15*	0.4	0.001**	0.7	0.182	0.51	0.001**
*fabp6*	2.43	0.053	0.62	0.015*	4.60	0.046*
*acsl5*	1.96	0.016*	1.06	0.132	3.46	0.012*
*cd36*	7.09	0.001**	4.40	0.007**	0.69	0.204
*gpat2*	1.22	0.023*	2.42	0.305	0.60	0.274
*pparγ*	1.54	0.236	1.09	0.771	0.35	0.03*
*acox1*	1.48	0.4	0.81	0.664	0.56	0.077
*cpt1b*	1.06	0.892	1.57	0.294	0.46	0.079
*plcg2*	1.01	0.968	0.51	0.093	1.13	0.699
*cpt1a*	0.98	0.936	0.82	0.752	0.66	0.866
*cyp51*	0.92	0.834	0.35	0.009**	1.37	0.49
*srebp*	0.90	0.408	2.62	0.302	1.27	0.276
*acc*	0.87	0.435	0.8	0.384	0.61	0.104
*acly*	0.85	0.153	0.51	0.058	0.49	0.067
*atgl*	0.71	0.419	0.8	0.072	1.35	0.025*


*^*a*^CF, Conventional zebrafish; GF, Germ-free zebrafish; CN, zebrafish without the exposure to antibiotics; AN, antibiotics-treated zebrafish. ^*b*^Full name of genes: *apoa4*: apolipoprotein A-IV b; *hsl:* hormone-sensitive lipase; *slc2a1a*: solute carrier family 2, facilitated glucose transporter member 1; *lss*: lanosterol synthase (2,3-oxidosqualene-lanosterol cyclase); *fabp6:* fatty acid binding protein 6; *acsl5*: acyl-CoA synthetase long-chain family member; *cd36*: cluster of differentiation 36, also known as platelet glycoprotein 4, fatty acid translocase; *gpat*: glycerol-3-phosphate acyltransferase; *pparγ*: peroxisome proliferator-activated receptor gamma; *cox15*: cytochrome c oxidase subunit; *acox1*: peroxisomal acyl-coenzyme A oxidase 1; *cpt1b:* carnitine palmitoyl transferase I b; *plcg2*: 1-phosphatidylinositol-4,5-bisphosphate phosphodiesterase gamma-2; *cpt1a:* carnitine palmitoyl transferase I a; *cyp51*: cytochrome P450, family 51; *srebp*: sterol regulatory element-binding proteins; *acc*: acetyl-CoA carboxylase; *acly*: ATP citrate lyase; *dgat2*: diacylglycerol O-acyltransferase 2; *atgl:* adipose triglyceride lipase. ^*c*^Fold change is the ratio of CF versus GF, CN versus AN. ^∗^*p* < 0.05, ^∗∗^*p* < 0.01.*

### Genes That Showed Organ-Specific Response to the Presence of Bacteria

The second cluster involves some genes which showed consistent alteration responses to the existence of intestinal bacteria in gut or liver, suggesting the functions of these genes are more organ-specific. For example, we found *acsl5* was upregulated in conventional zebrafish. The expression of *acsl5* has been suggested to play a role in liver cell lines to promote fatty acid uptake, leading to either increased storage (Mashek et al., 2006) or β-oxidation (Zhou et al., 2007). This result suggests that the expression of *acsl5* may be related to intestinal microbiota. The genes, *cd36* and *gpat2*, are responsible for importing fatty acids inside cells and glycerolipid biosynthesis, respectively. Considering the lower substrate utilization efficiency in germ-free or antibiotics-treated zebrafish (Semova et al., 2012), higher expression levels of these genes were found in conventional zebrafish, especially in the liver.

### Genes That Were Influenced by Antibiotics Treatment

In addition to the above genes, we also found some genes remained unchanged between the conventional and germ-free zebrafish or showed inconsistent pattern towards intestinal microbiota in these two models, indicating the expression of these genes are related to other factors instead of intestinal microbiota. For example, *pparγ* and *srebp* were upregulated after bromochloromethane treatment in Sprague–Dawley rat (Su et al., 2015) and *pparγ* was influenced by antibiotics treatment (Szkudlarek-Mikho et al., 2012). The expression of *acox1* was upregulated in mice receiving probiotic treatment (Park et al., 2013) and further it was influenced by an anti-bacterial agent, triclosan (Wu et al., 2014). Consistent with this study, we also found *acox1* was upregulated in the intestine of antibiotic-treated zebrafish, although no significant difference was detected. The genes, *cyp51* and *acly*, are potential drug targets (Barrow et al., 1997; Dauchy et al., 2016). Our results suggested these genes may be more influenced by antibiotics treatment instead of intestinal bacteria, which is in agreement with previous findings.

Our results showed that antibiotics decreased the expression of *dgat2* in both liver and intestine. It has been found that antibiotic treatment induced endoplasmic reticulum stress, which substantially inhibited the expression of lipogenic genes including *dgat2* (Koc et al., 2015). Accordingly, we hypothesize that this gene is more related to antibiotics treatment. In the present study, the expression level of *atgl* gene did not show any change between the conventional and germ-free zebrafish but it was changed in the liver and intestine when zebrafish were treated with antibiotics. *Atgl* mediated the antibiotics stimulated reproduction in the brown planthopper (Jiang et al., 2016), which is consistent to the present results. The two carnitine palmitoyltransferases, *cpt1a* and *cpt1b*, involved in mitochondrial fatty acid oxidation were upregulated in germ-free zebrafish (Rawls et al., 2004), while in the present study, we did not find a similar trend. It has been shown that, the direct effects of antibiotics on host tissues and antibiotic-resistant microbe primarily inhibit mitochondrial genes expression (Morgun et al., 2015). Therefore, whether the expression of these genes is related to intestinal or antibiotics treatment remains unclear.

### The Advantages and Disadvantages of Germ-Free and Antibiotics-Treated Zebrafish Models

In this study, we used germ-free and antibiotic-treated zebrafish to identity the relationship between the intestinal microbiota and host metabolism. Antibiotic treatment and germ-free are very popular for identifying the function of commensals in mammals and great progress has been made. For example, it has been found that antibiotic treatment in mice notably decreased the expression level of RegIIIγ, which was supposed to be induced by intestinal commensals (Brandl et al., 2008). However, the combination of intestinal transcriptome and metagenomic analysis showed that antibiotic-induced alteration can be attributed to three factors: depletion of commensals, direct influence on host tissues, and the effects of antibiotics-resistant bacteria (Morgun et al., 2015). Therefore, it is difficult to identify the exact role of intestinal microbiota solely based on antibiotics-treated animal model. Germ-free animal model is more suitable for microbe-host interaction identification. Germ-free zebrafish was established in 2008 (Pham et al., 2008). Indeed, it has broadened our knowledge on microbiota functions in host metabolism. However, the small size of germ-free zebrafish larvae hinders the discovery of the various responses from different organs due to intestinal commensals. In the present study, we combined these two models and selected several genes related to intestinal microbiota in zebrafish.

## Conclusion

This comparative study revealed that genes associated with lipid catabolism and cholesterol synthesis were upregulated in germ-free or antibiotics-treated zebrafish, while those related to lipid absorption or biosynthesis increased in conventional zebrafish. We also compared the advantages and disadvantages of germ-free zebrafish or antibiotics-treated zebrafish in revealing the intestinal microbiota function in fish nutritional or metabolism research. This study provides valuable information for the use of the two models in future research to unveil the energy homeostasis and lipid metabolism in fish.

## Author Contributions

MZ and Z-YD designed the research. YS, HR, and YhS conducted the research. WZ and FQ analyzed the data. MZ, YS, and SL wrote the manuscript. All the authors read and approved the final manuscript.

## Conflict of Interest Statement

The authors declare that the research was conducted in the absence of any commercial or financial relationships that could be construed as a potential conflict of interest.

## Footnotes

1 http://www.bioinformatics.babraham.ac.uk/projects/fastqc/

2 http://ccb.jhu.edu/software/tophat/index.shtml

3 http://eggnog.embl.de

4 http://htseq.readthedocs.io/en/release_0.10.0/

5 http://bioconductor.org/packages/release/bioc/html/DESeq.html
