# Supplementary material for: The Presence or Absence of Intestinal Microbiota Affects Lipid Deposition and Related Genes Expression in Zebrafish (Danio rerio)
Source: Front Microbiol. 2018 May 29;9:1124. doi: 10.3389/fmicb.2018.01124 (PMC5987169; doi:10.3389/fmicb.2018.01124)
Supplement: Supplementary file 1 [file Data_Sheet_1.docx]

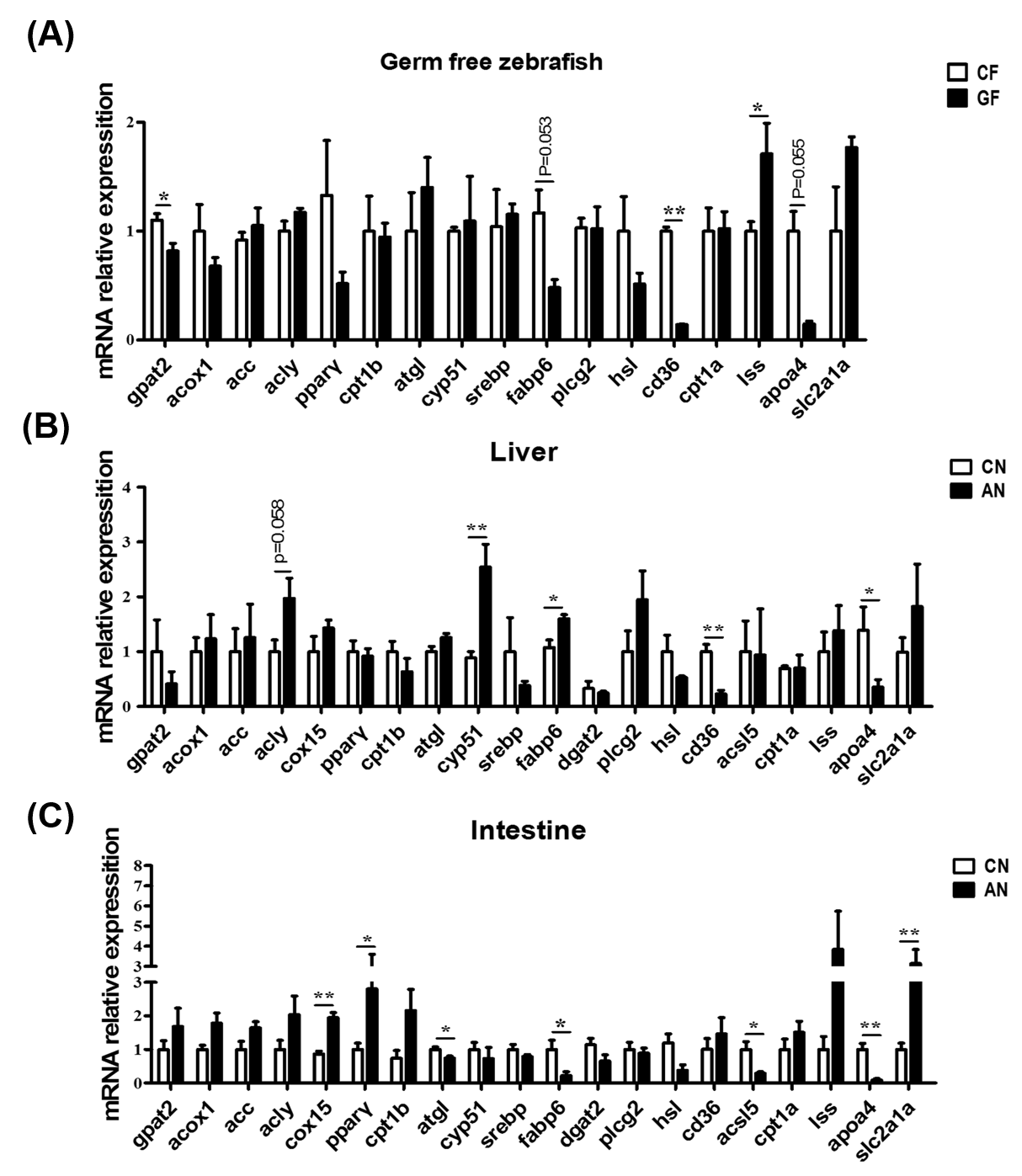


Figure S1. Representative gene expression level in the liver (A) or gut (B) in control and antibiotic-treated zebrafish. Data are presented as mean ± SEM. The expression levels normalized to CF. Significant difference was determined by student’s t test,* P≤0.05, ** P<0.01.

**Table S1** Formulation and nutrition composition of experimental diet

| Component (g/kg) | Diet |
| --- | --- |
| Casein | 360 |
| Gelation | 80 |
| Corn starch | 324.65 |
| Soybean oil | 120 |
| Mixed vitamin^1^ | 10 |
| Mixed minerals^2^ | 40 |
| Carboxy methyl cellulose (CMC) | 30 |
| Cellulose | 28.6 |
| Choline chloride | 5 |
| Butylated hydroxytoluene (BHT) | 0.25 |
| [Phagostimulant](http://www.so.com/link?url=http%3A%2F%2Fdict.youdao.com%2Fsearch%3Fq%3Dphagostimulant%26keyfrom%3Dhao360&q=%E8%AF%B1%E9%A3%9F%E5%89%82%E8%8B%B1%E6%96%87&ts=1516716778&t=6f5f21666dab88f9656b0df87592e2f) | 1.50 |
| Total(g) | 1000 |
| Total fat(%) | 12 |
| Total protein(%) | 44 |

**^1^** mixed vitamin ， (mg or IU/kg)： 500,000 I.U. (international units) Vitamin A， 50,000 I.U. VitaminD3， 2500 mg Vitamin E， 1000 mg Vitamin K3， 5000 mg Vitamin B1， 5000 mg Vitamin B2，5000 mg Vitamin B6， 5000 μg Vitamin B12， 25,000 mg Inositol， 10,000 mg Pantothenic acid，100,000 mg Cholin， 25,000 mg Niacin， 1000 mg Folic acid， 250 mg Biotin 10,000 mg Vitamin C

**^2^** mixed minerals， (g/kg)： 314.0 g CaCO3， 469.3g KH2PO4， 147.4 g MgSO 4·7H2O， 49.8 g NaCl， 10.9 g Fe(II)gluconate， 3.12 g MnSO4 ·H2O， 4.67 g ZnSO4·7H2O， 0.62 g CuSO4·5H2O，0.16 g KJ， 0.08 g CoCl 2 ·6H 2 O， 0.06 g NH 4 molybdate， 0.02 g NaSeO 3

**Table S2** Primers used for qRT-PCR expression analysis

| **Gene** |  | **Primer Sequence (5’-3’)** |
| --- | --- | --- |
| ef1α | F | CCCCTGGACACAGAGACTTCATC |
|  | R | AATTTCAGGATGGCACGGTGA |
| lss | F | GGCGAAGCAGATGGAGGATGGA |
|  | R | CAGCAGGTGTTGTGGATCTGAGAG |
| srebp | F | ACGCCTGTTGTTGTTGTTGT |
|  | R | GTCCAGAGTGTCCAGAGAAGT |
| atgl | F | AACTCATCCAGGCTCTCAT |
|  | R | TTCCACCATCCACATAACG |
| fas | F | ATGGCTCGCACTCTTATG  ATGGCTCGCACTCTTATG |
|  | R | CGGTGAACTGTTGGATGA |
| cyp51 | F | GCAGAGAAGATACCGAGAA |
|  | R | TCATCGTCACTCAGAGGA |
| hsl | F | CGGCAAGGACAGGACAGT |
|  | R | GCATGGAGAAAGAGGAGCT |
| magl | F | TTGTTCTGTCGTTACTGGGAGC |
|  | R | CTGTGAGCAATGTCAGCGTAT |
| cd36 | F | TCACGTCACTGGAGAACTCG |
|  | R | TCATGTCGCCCACCGGGATG |
| fabp6 | F | CCCGCTCTTCTTCTCCGCTCA |
|  | R | GTCACGGCCCTTTGCGATGACA |
| cpt1a | F | CATCCTTAGGCCTGCTCTTCAAA |
|  | R | ACCATGACACCCCCAACTAACAT |
| cpt1b | F | CCTCCATGGGCACGATTGATAA |
|  | R | GCAAACAGGATGGCACTCAACA |
| acc | F | GCGTGGCCGAACAATGGCAG |
|  | R | GCAGGTCCAGCTTCCCTGCG |
| acly | F | ACTGCAGAAACCGACTGGGAGA |
|  | R | CAGCTTTCCTCTGCGCTTTATCA |
| gpat2 | F | GTCGGACAGTGCTGTCACCAAT |
|  | R | ACACGTCAAGTTCTGAAAGCCCA |
| dgat2 | F | ACGCATAACCTGCTTCCC |
|  | R | TCCTGTGGCTTCTGTCCC |
| pparα | F | TGCTGGACTACCAGAACTGTGACA |
|  | R | TGCTGGCTGAGAACACTTCTGAG |
| pparγ | F | AATTCGCCAAGAGCATCCCG |
|  | R | ATGAGCGGAGAAATCATGATGATC |
| apob | F | TGAGAATGGGGCTTTGGGTC |
|  | R | TATGTCCTGAGGGACGGGAA |
| cpt2 | F | ATTTGCACAAGAGTATCGTCCCG |
|  | R | GCTGCCAAATACCTCCGTATTGTA |
| slc2a1a | F | GGACGAGAGCAGACAGATGATGAG |
|  | R | GCAGCATGATGGCGATGAAGATG |
| cox15 | F | TGGTACATGGTGAAGAGCGGTCT |
|  | R | CAGCAGAGCGGAGCCTAGATGA |
| acsl5 | F | GCAGGGCATTATCAGGAACGACAG |
|  | R | GCTCCAGTCACCATCACTCTCACA |
| plcg2 | F | GCGGAGGACTATCTAATGCGGATC |
|  | R | AAGCCTTCGCCTCTGAATGTGATG |
| apoa4 | F | ACAGACTAAAAGTGAAGACCATGAAG |
|  | R | GGATCAAATATGTCAAATTTCGAAGTTTGTG |
| acox1 | F | AGTGCGGAAGAGTGCACAG |
|  | R | ACACTGGCTTTTTAAGGTGGGT |

**Table S3** Summary for the RNA-seq outcomes of conventional and germ-free zebrafish

| Sample | Raw Reads | Clean Reads | Q20(%) | Q30(%) | GC (%) | Mapped reads | Uniquely Mapped  Percent % |
| --- | --- | --- | --- | --- | --- | --- | --- |
| CF^a^ | 35,824,498 | 35,291,992 | 94.85% | 91.02% | 54.85% | 31,309,478 | 80.96% |
| GF | 32,882,790 | 32,423,470 | 95.07% | 90.82% | 53.23% | 27,359,377 | 96.26% |

^a^ CF: Conventional zebrafish, GF: Germ-free zebrafish.

**Table S4.** The main function of the selected genes and their possible relationship with the intestinal microbiota or antibiotic treatment.

| **Gene** | **Function or their relationship with microbiota or antibiotic treatment** | **Reference** |
| --- | --- | --- |
| *apoa4* | Intestinal lipid absorption; Energy storage; regulated by LPS. | (Wang et al., 2012) (Dandekar et al., 2016) |
| *slc2a1a* | Glucose transporter | (Swartz et al., 2012) |
| *lss* | lanosterol synthase | (Zhong et al., 2015) |
| *fabp* | Assess the gut permeability indirectly | (Donnadieu-Rigole et al., 2018), |
| *acsl5* | Promote fatty acid uptake | (Mashek et al., 2006) (Zhou et al., 2007) |
| *cd36* | Import fatty acids inside cells; | (Abumrad et al., 1993) |
| *gpat2* | Glycerolipid biosynthesis | (Wang et al., 2007) |
| *PPARγ* | Influenced by antibiotics treatment | (Szkudlarek-Mikho et al., 2012) |
| *acox1* | Influenced by antibiotics treatment | (Wu et al., 2014) |
| *cyp51* | Potential drug targets | (Dauchy et al., 2016) |
| *acly* | Potential drug targets | (Barrow et al., 1997) |
| *dgat2* | Catalyzes a reaction which produces TAG from DAG and fatty acyl-CoA, | (Koc et al., 2015) |
| *atgl* | Mediated the antibitoics jinggangmycin-stimulated reproduction in the brown plaihooper | (Jiang et al., 2016) |
| *cpt1a/1b* | Mitochondrial fatty acid oxidation | (Cohen et al., 1998) |

**Reference**

Abumrad, N.A., el-Maghrabi, M.R., Amri, E.Z., Lopez, E., and Grimaldi, P.A. (1993). Cloning of a rat adipocyte membrane protein implicated in binding or transport of long-chain fatty acids that is induced during preadipocyte differentiation. Homology with human CD36. *J .Biol.Chem.* 268**,** 17665-17668.

Barrow, C.J., Oleynek, J.J., Marinelli, V., Sun, H.H., Kaplita, P., Sedlock, D.M., et al. (1997). Antimycins, inhibitors of ATP-citrate lyase, from a Streptomyces sp. *J. Antibiot .(Tokyo)* 50**,** 729-733.

Cohen, I., Kohl, C., McGarry, J.D., Girard, J., and Prip-Buus, C. (1998). The N-terminal domain of rat liver carnitine palmitoyltransferase 1 mediates import into the outer mitochondrial membrane and is essential for activity and malonyl-CoA sensitivity. *J. Biol. Chem.* 273**,** 29896-29904.

Dandekar, A., Qiu, Y.N., Kim, H., Wang, J.M., Hou, X., Zhang, X.B., et al. (2016). Toll-like Receptor (TLR) Signaling Interacts with CREBH to Modulate High-density Lipoprotein (HDL) in Response to Bacterial Endotoxin. *J. Biol. Chem.* 291**,** 23149.

Dauchy, F.A., Bonhivers, M., Landrein, N., Dacheux, D., Courtois, P., Lauruol, F., et al. (2016). Trypanosoma brucei CYP51: Essentiality and Targeting Therapy in an Experimental Model. *PLoS. Negl. Trop. Dis.* 10**,** e0005125. doi: 10.1371/journal.pntd.0005125PNTD-D-16-00870 [pii].

Donnadieu-Rigole, H., Pansu, N., Mura, T., Pelletier, S., Alarcon, R., Gamon, L., et al. (2018). Beneficial Effect of Alcohol Withdrawal on Gut Permeability and Microbial Translocation in Patients with Alcohol Use Disorder. *Alcohol. Clin. Exp. Res.* 42**,** 32-40.

Jiang, Y.P., Li, L., Liu, Z.Y., You, L.L., Wu, Y., Xu, B., et al. (2016). Adipose triglyceride lipase (Atgl) mediates the antibiotic jinggangmycin-stimulated reproduction in the brown planthopper, Nilaparvata lugens Stal. *Sci. Rep.* 6**,** 18984. doi: 10.1038/srep18984srep18984 [pii].

Koc, M., Mayerova, V., Kracmerova, J., Mairal, A., Malisova, L., Stich, V., et al. (2015). Stress of endoplasmic reticulum modulates differentiation and lipogenesis of human adipocytes. *Biochem. Biophys. Res. Commun.* 460**,** 684-690.

Mashek, D.G., McKenzie, M.A., Van Horn, C.G., and Coleman, R.A. (2006). Rat long chain acyl-CoA synthetase 5 increases fatty acid uptake and partitioning to cellular triacylglycerol in McArdle-RH7777 cells. *J. Biol. Chem.* 281**,** 945-950.

Swartz, T.D., Duca, F.A., de Wouters, T., Sakar, Y., and Covasa, M. (2012). Up-regulation of intestinal type 1 taste receptor 3 and sodium glucose luminal transporter-1 expression and increased sucrose intake in mice lacking gut microbiota. *Brit. J.Nutr.* 107**,** 621-630.

Szkudlarek-Mikho, M., Saunders, R.A., Yap, S.F., Ngeow, Y.F., and Chin, K.V. (2012). Salinomycin, a polyether ionophoric antibiotic, inhibits adipogenesis. Biochem. Bioph. Res. Co. 428, 487-493.

Wang, F., Kohan, A.B., Kindel, T.L., Corbin, K.L., Nunemaker, C.S., Obici, S., et al. (2012). Apolipoprotein A-IV improves glucose homeostasis by enhancing insulin secretion. *Proc. Natl. Acad. Sci. U. S. A.* 109**,** 9641-9646.

Wang, S., Lee, D.P., Gong, N., Schwerbrock, N.M., Mashek, D.G., Gonzalez-Baro, M.R., et al. (2007). Cloning and functional characterization of a novel mitochondrial N-ethylmaleimide-sensitive glycerol-3-phosphate acyltransferase (GPAT2). *Arch. Biochem. Biophys.* 465(2)**,** 347-358.

Wu, Y., Wu, Q., Beland, F.A., Ge, P., Manjanatha, M.G., and Fang, J.L. (2014). Differential effects of triclosan on the activation of mouse and human peroxisome proliferator-activated receptor alpha. *Toxicol. Lett.* 231**,** 17-28.

Zhong, C.Y., Sun, W.W., Ma, Y., Zhu, H., Yang, P., Wei, H., et al. (2015). Microbiota prevents cholesterol loss from the body by regulating host gene expression in mice. *Sci. Re.p* 5**,** 10512. doi: 10.1038/srep10512srep10512 [pii].

Zhou, Y., Abidi, P., Kim, A., Chen, W., Huang, T.T., Kraemer, F.B., et al. (2007). Transcriptional activation of hepatic ACSL3 and ACSL5 by oncostatin M reduces hypertriglyceridemia through enhanced beta-oxidation. *Arterioscl. Throm. Vas.* 27**,** 2198-2205.
